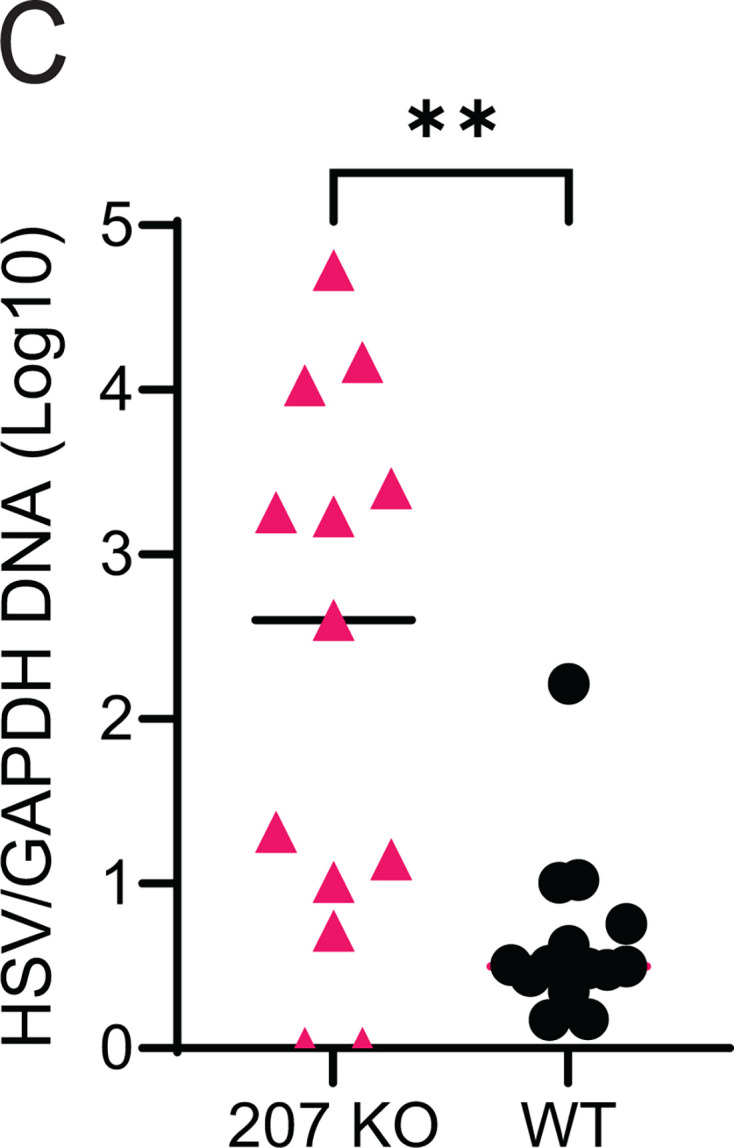# Correction for Enya et al., “IFI207 promotes antiviral responses by modulating STING ubiquitination and degradation”

**DOI:** 10.1128/mbio.01520-26

**Published:** 2026-07-10

**Authors:** Takuji Enya, Wenming Zhao, Geetanjali, Bin He, Susan R. Ross

## AUTHOR CORRECTION

Volume 17, no. 6, e00612-26, 2026, https://doi.org/10.1128/mbio.00612-26. Figure 5: Panel C should appear as shown in this correction. Due to an error during figure assembly, the incorrect graph was inserted. The figure legend and raw data in the Mendeley file remain unchanged. The results and conclusions of the study remain unaffected. We sincerely apologize for this error.

**Fig 5:**