# Supplementary material for: IFI207 promotes antiviral responses by modulating STING ubiquitination and degradation
Source: mBio. 2026 May 11;17(6):e00612-26. doi: 10.1128/mbio.00612-26 (PMC13251359; doi:10.1128/mbio.00612-26)
Supplement: Supplemental Figures — Fig. S1 to S4. [file mbio.00612-26-s0001.pdf]

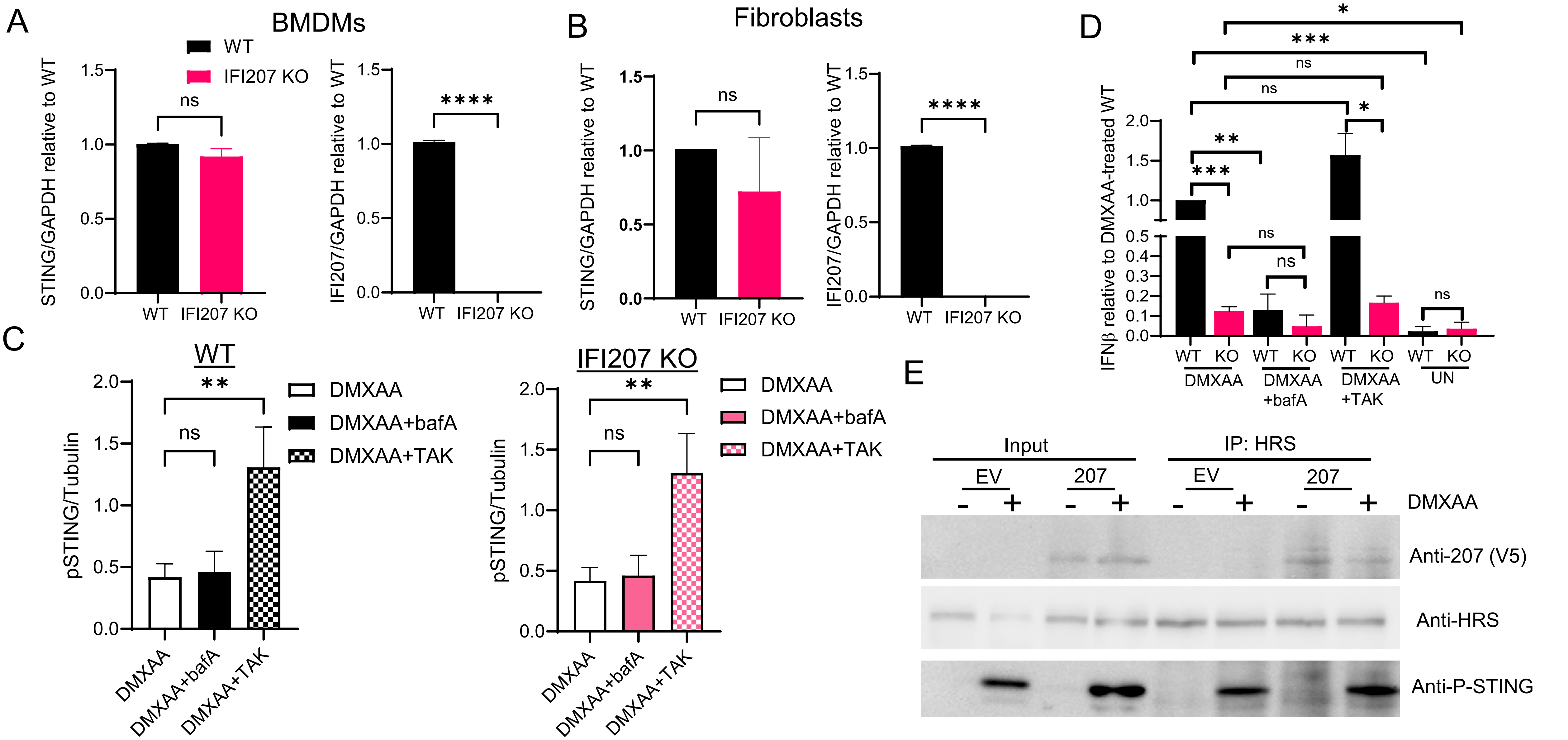

Suppl. Fig. 1. IFI207 promotes STING signaling. BMDMs (A) and fibroblasts (B) from IFI207 KO and wild-type mice were treated with 100 µg/mL DMXAA and RNA was examined by RT-qPCR for the levels of STING and GAPDH. Quantification of 3 independent experiments ± SD is shown. Paired t-tests were used to determine significance. ****, *P*≤0.007. (C) Comparison of p-STING levels in DMXAA, DMXAA+bafA and DMXAA-treated macrophages from WT and IFI207 KO mice 3 hr post-DMXAA treatment. Replotted data from Fig. 1A. One-way ANOVA was used to determine significance. **, *P*≤0.005. (D) IFNα levels produced in WT and IFI207 KO fibroblasts was measured by ELISA at 2 hr post-DMXAA treatment. UN, untreated. Quantification of 3 independent experiments ± SD is shown. Two-tailed t-tests were used to determine significance. *, P≤0.05, **, P≤0.01, ***, P≤0.001.(E) HRS co-immunoprecipitates with pSTING.

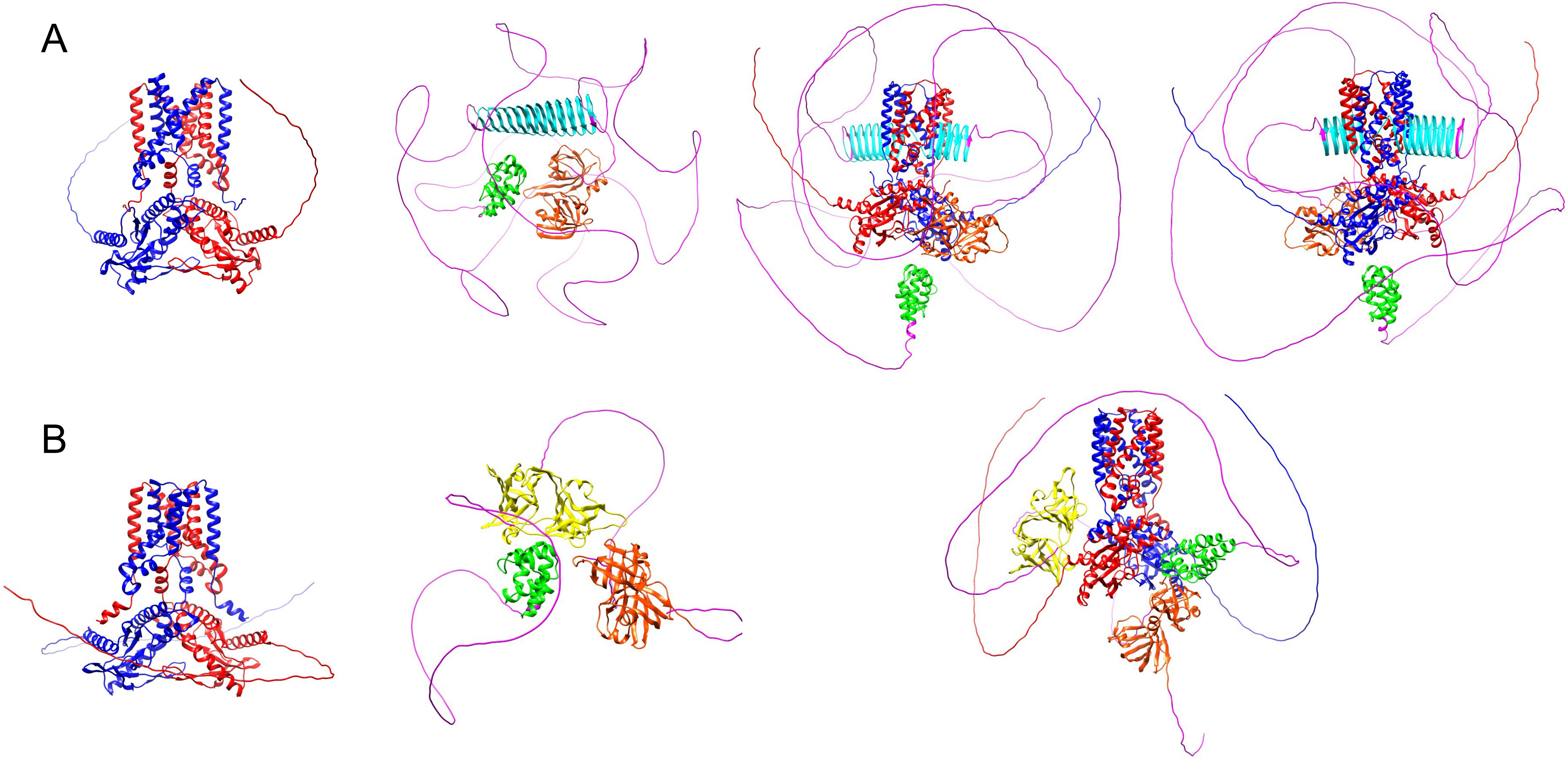

Suppl. Fig. 2. A) AlphaFold predicted structure of the mouse STING dimer (red and blue) and IFI207 (magenta). The mouse STING/IFI207 complex is shown in both front and back. The N terminal pyrin domain is green, the C terminal HINB domain is orange, the repeat region is cyan. B) AlphaFold predicted structure of the human STING dimer (red and blue) and IFI16 (magenta). Pyrin and HINB domains colored as in A; the HINA domain is yellow. Structures were downloaded from AlphaFold Server (https://alphafoldserver.com/) (Abramson, 2024). UCSF Chimera was used to generate images (https://www.rbvi.ucsf.edu/chimera) (Petterson et al., 2004).

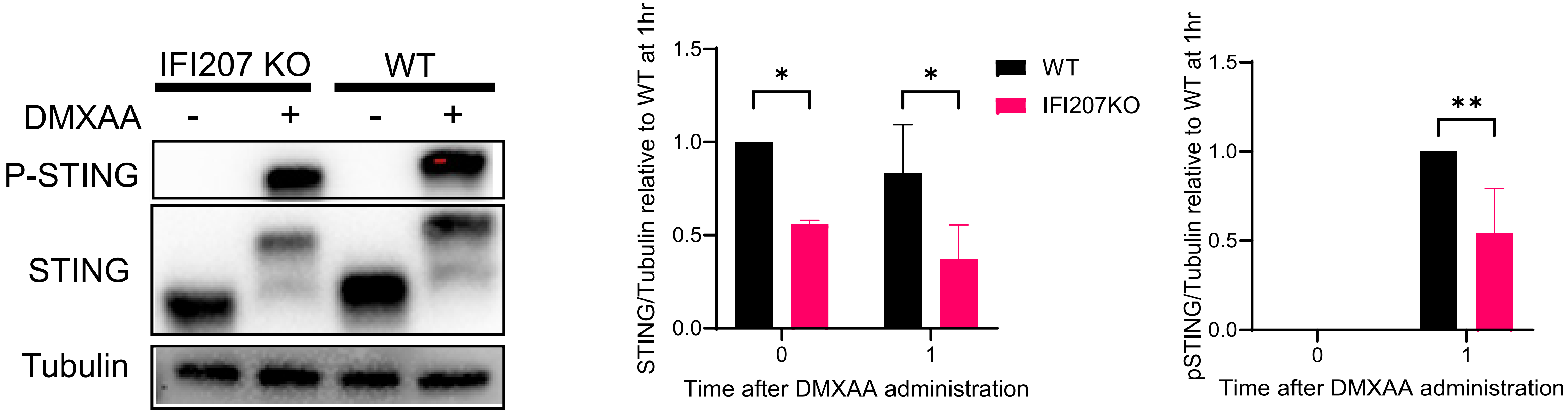

Suppl. Fig. 3. IFI207 stabilizes STING in BMDCs. BMDCs from IFI207 KO and wild-type mice were treated with 100 µg/ml DMXAA and STING and pSTING protein levels were examined by western blot at 1 hr post-treatment. Shown to the right is quantification of 3 independent experiments $\pm$ SD. Two-way ANOVA was used to determine significance. *, $P \leq 0.02$; **, $P \leq 0.004$.

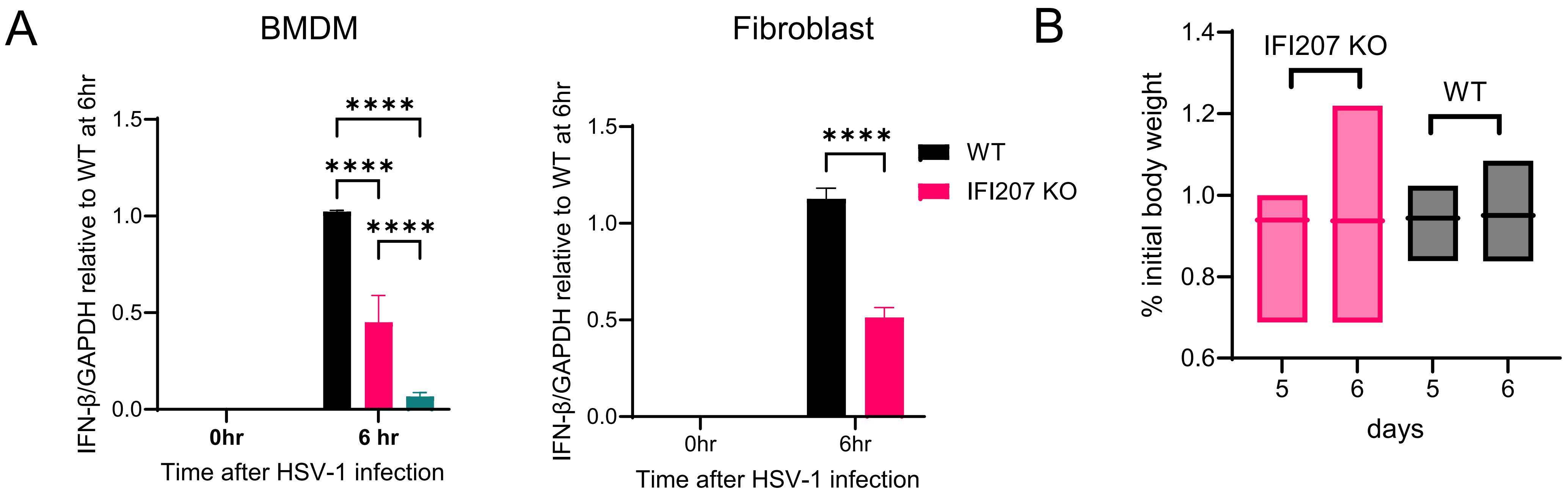

Suppl. Fig. 4. IFI207 promotes STING signaling in response to virus infection. A) BMDMs and fibroblasts from the indicated mice were infected HSV-1 for 6 hr (MOI=5), IFNβ mRNA levels were quantified by RT-qPCR. Shown is the average of 3 experiments. Two-way ANOVA was used to determine significance. ****, P≤0.0001. B) average weight loss in IFI207 KO and wild type mice at 5- and 6-days post-infection.
